# Molecular and immunological tools for the evaluation of the cellular immune response in the neotropical monkey *Saimiri sciureus*, a non-human primate model for malaria research

**DOI:** 10.1186/s12936-015-0688-1

**Published:** 2015-04-18

**Authors:** Evelyn KP Riccio, Lilian R Pratt-Riccio, Cesare Bianco-Júnior, Violette Sanchez, Paulo RR Totino, Leonardo JM Carvalho, Cláudio Tadeu Daniel-Ribeiro

**Affiliations:** Laboratório de Pesquisa em Malária, Instituto Oswaldo Cruz, Fiocruz, Avenida Brasil 4365, Pavilhão Leônidas Deane, Salas 513-517, 5° andar Manguinhos, Rio de Janeiro, RJ CEP: 21040-900 Brazil; Centro de Pesquisa, Diagnóstico e Treinamento em Malária (CPD-Mal), Fiocruz, Reference Centre for Malaria in the Extra-Amazonian Region for the Secretary for Health Surveillance, Ministry of Health, Rio de Janeiro, RJ Brazil; Present address: Research Department, Sanofi Pasteur, Lyon, France

**Keywords:** *Saimiri sciureus*, Immune response, Malaria, Cytokines, Cellular response

## Abstract

**Background:**

The neotropical, non-human primates (NHP) of the genus *Saimiri* and *Aotus* are recommended by the World Health Organization as experimental models for the study of human malaria because these animals can be infected with the same *Plasmodium* that cause malaria in humans. However, one limitation is the lack of immunological tools to assess the immune response in these models. The present study focuses on the development and comparative use of molecular and immunological methods to evaluate the cellular immune response in *Saimiri sciureus*.

**Methods:**

Blood samples were obtained from nineteen uninfected *Saimiri*. Peripheral blood mononuclear cells (PBMC) from these animals and splenocytes from one splenectomized animal were cultured for 6, 12, 18, 24, 48, 72 and 96 hrs in the presence of phorbol-12-myristate-13-acetate and ionomycin. The cytokine levels in the supernatant were detected using human and NHP cytometric bead array Th1/Th2 cytokine kits, the Bio-Plex Pro Human Cytokine Th1/Th2 Assay, enzyme-linked immunosorbent assay, enzyme-linked immunospot assays and intracellular cytokine secretion assays. Cytokine gene expression was examined through TaqMan® Gene Expression Real-Time PCR using predesigned human gene-specific primers and probes or primers and probes designed based on published *S. sciureus* cytokine sequences.

**Results:**

The use of five assays based on monoclonal antibodies specific for human cytokines facilitated the detection of IL-2, IL-4 and/or IFN-γ. TaqMan array plates facilitated the detection of 12 of the 28 cytokines assayed. However, only seven cytokines (IL-1A, IL-2, IL-10, IL-12B, IL-17, IFN-β, and TNF) presented relative expression levels of at least 70% of the gene expression observed in human PBMC. The use of primers and probes specific for *S. sciureus* cytokines facilitated the detection of transcripts that showed relative expression below the threshold of 70%. The most efficient evaluation of cytokine gene expression, in PBMC and splenocytes, was observed after 6–12 hrs of culture, except for LTA in PBMC, whose expression was best analysed after 24 hrs of culture.

**Conclusions:**

Real-time PCR facilitates the analysis of a large number of cytokines altered during malaria infection, and this technique is considered the best tool for the evaluation of the cellular immune response in *S. sciureus*.

## Background

Research involving non-human primates (NHP) has played a critical role in many of the medical and scientific advances in the last decade. NHP are used because of the similarities of these animals to humans in terms of physiology, neuroanatomy, reproduction, development, cognition, and social complexity. The close phylogenetic relationship of NHP to humans also makes these animals excellent models for the study of a range of biological phenomena. These similarities between humans and NHP suggest that there is greater validity of the data obtained from primate models compared to other animal models, and experiments conducted using these models might address questions that cannot be addressed using other species.

For several infectious diseases, NHP are the only animal species susceptible to the infectious agent that threatens human populations, and proof-of-concept of candidate vaccines can therefore only be studied in these species. The choice of the NHP model depends on the nature of the pathogen. Hepatitis virus has a host range restricted to humans and some NHP, including *Callithrix jaccus*, *Macaca mulatta*, *Macaca fascicularis* and chimpanzee, making these models valuable for studies on hepatitis [1-5]. In AIDS research, NHP are used to investigate the mechanisms underlying immune system regulation and disease pathogenesis and to optimize immunization strategies and vaccine safety and immunogenicity [6-8]. Other infectious agents for which NHP have been valuable for vaccine research include influenza virus, *Trypanosoma cruzi*, *Mycobacterium*, flavivirus, poliovirus, *Bacillus anthracis*, *Helicobacter pylori*, Ebola virus, and *Plasmodium* species [9].

Experimental models have been used from the inception of malariology and have provided important insights into the mechanisms underlying *Plasmodium* diseases [10,11]. Many studies on malaria have used rodent or NHP experimental models, which have been long-standing tools for malaria immunology and pathogenesis studies [12]. There is, however, no animal model as reliable as the NHP for studying the basic mechanisms of human diseases [13]. In addition, recent studies have reported the natural infection of NHP, such as bonobos and chimpanzees, with human *Plasmodium* or plasmodial ancestors [14-16].

The NHP of the genera *Aotus* and *Saimiri* are the experimental models recommended by the World Health Organization for research in malaria [17-20] because these NHP develop a reproducible parasitaemia when inoculated with the blood stages or even sporozoites of the human plasmodial species *Plasmodium falciparum* and *Plasmodium vivax*, making these animals particularly useful in preclinical trials of potential malaria vaccines and for studies on pathogenesis [21-25]. In addition, strains adapted to NHP have been used in studies to test schizonticidal anti-malarial drugs, malaria vaccine studies examining both pre-erythrocytic candidate antigens and transmission-blocking vaccines and *P. vivax* biology and genetic studies in both invertebrate mosquito vectors and primate vertebrate hosts [26,27]. Preclinical evaluation in these experimental models can provide valuable information on the immunogenicity, efficacy and safety of a variety of formulations, facilitating a more refined selection of the most appropriate formulations for evaluation in humans.

Although the NHP model might offer many advantages for the study of malaria, one important limitation is the lack of specific reagents and immunological tools for the reliable evaluation of primate immune responses. In some cases, immunological reagents for human molecules can be used, although sensitivity is typically low [28,29]. Few cytokine studies have been performed in *Saimiri* [30-32], although research efforts have been focused on sequencing individual cytokine genes of interest. Moreover, researchers have also attempted to develop molecular tools to measure the mRNA expression of IL-2, IL-5, IL-6, IL-10, IL-12, LTA, TNF, and IFN-γ [30,33]. The present study focused on the development and comparative use of molecular and immunological methods to monitor the cellular immune response in *Saimiri sciureus* monkeys, with the aim of providing information for future immunization studies involving *P. falciparum* vaccine candidates.

## Methods

### Animals and legal bioethics aspects

Nineteen clinically healthy NHP of the *Saimiri sciureus* species from the breeding colony at the Department of Primatology (CECAL)/Fiocruz in Rio de Janeiro, Brazil were sampled and used in different assays. These animals were young, ranging between four and eight years of age. The experiments involving *Saimiri* were reviewed and approved through the Ethics Committee on Animal Use of Fiocruz (CEUA, Fiocruz, Rio de Janeiro, Brazil protocol P-391/07) and conducted in accordance with the requirements of the laboratory biosafety rules (License n° L-0062/08).

### Tissue sampling, isolation and culture of cells

The animals were anaesthetized with a combination of 0.1 ml midazolan and 0.4 ml ketamine. Blood samples were collected via femoral venipuncture, and cells were obtained from 4 ml heparinized venous samples obtained from each individual. The peripheral blood mononuclear cells (PBMC) were separated through density gradient centrifugation using Ficoll-Hypaque (Sigma), washed twice in phosphate-buffered saline (PBS) (Sigma), resuspended and cultured in RPMI medium containing 15 mM glutamine (Gibco), 10 mM Hepes, 200 U/ml penicillin (Gibco), 200 μg/ml streptomycin (Gibco), 3 mg/ml gentamicin (Sigma) and 2 g/l sodium bicarbonate (Sigma) supplemented with 10% inactivated foetal calf serum (FCS) (Imunoquímica). The cells (2.5 × 10^5^ cells/well) were activated with a combination of 50–100 ng/ml phorbol 12-myristate 13-acetate (PMA-Sigma) and 250–500 ng/ml ionomycin (Sigma) and cultured at 37°C in 5% CO_2_. The cells and supernatant were collected after 6, 12, 18, 24, 48, 72 and 96 hrs. Subsequently, the cells were resuspended in RNAlater solution (Ambion Life Technologies), incubated at 4°C overnight and subsequently stored at −20°C until RNA extraction for the analysis of gene expression using real-time PCR. The supernatant was stored at −80°C prior to the detection and quantification of cytokines. In all experiments, human blood samples from two healthy donors from the Laboratory for Malaria Research were collected and used as control.

One naive *Saimiri* monkey (PA51) was splenectomized, and the splenocytes were used in cellular assays. A trained veterinarian performed the splenectomy using ketamine (150 mg/kg) and xylazine (10 mg/kg) for anaesthesia in an aseptic surgical room. After spleen removal and vascular resection, the wound was sutured, and the animal was allowed to recover under the close supervision of trained staff. Immediately after removal, a piece of the spleen was conditioned in sterile RPMI 1640 medium supplemented with 10% FCS and gently fragmented using a cell strainer. The splenocytes were washed with RPMI 1640 medium supplemented with 10% FCS, and 3 ml of erythrocyte lysis buffer was added to the pellet and incubated for 5 min at 37°C. The splenocytes were cultured *in vitro* under mitogenic stimulus with PMA (Sigma) and ionomycin (Sigma) as previously described. The cells were collected after 6, 12, 18, 24, 48, 72 and 96 hrs of culture, resuspended in RNAlater solution (Ambion Life Technologies), incubated at 4°C overnight and subsequently stored at −20°C prior to RNA extraction for the analysis of gene expression using real-time PCR.

### Cytometric bead array (CBA)

The cytokine levels were assessed using two BD Cytometric Bead Array (CBA) Th1/Th2 Cytokine Kits (BD Bioscience). One human kit for the detection of IL-2, IL-4, IL-6, IL-10, TNF and IFN-γ and other NHP kit (rhesus, cynomolgus, baboon, or pigtailed macaque non-human primate species) for the simultaneous detection of six cytokines (IL-2, IL-4, IL-5, IL-6, TNF and IFN-γ) in the supernatant of PBMC cultures.

Samples from two *Saimiri* (V17 and V31) were used for the evaluation of human CBA kit and samples from three *Saimiri* (S8, S34 e S36) for the evaluation of NHP CBA kit. Blood samples obtained from one human healthy donor were used as control. The cytokine levels were determined in the test samples according to the manufacturer’s instructions. Briefly, 25 μl of the supernatant obtained from PBMC cultures was incubated for 2 hrs at room temperature (RT) with 25 μl of cytokine capture beads and 25 μl of PE detection reagent. After incubation, the samples were washed once with washing buffer. The supernatants were discarded, and the pellets were resuspended in 300 μl of washing buffer for analysis on a FACS Calibur flow cytometer (Becton Dickinson). The concentration of each cytokine in pg/ml was determined based on a standard curve generated using the recombinant cytokines provided in the kit. In human CBA kit, the lower limits of detection for IL-2, IL-4, IL-6, IL-10, TNF and IFN-γ were 2.6, 2.6, 3.0, 2.8, 2.8 and 7.1 pg/ml, respectively, while in NHP CBA kit, the lower limits of detection for IL-2, IL-4, IL-5, IL-6, TNF and IFN-γ were 3.6, 0.9, 0.3, 0.1, 0.4, 3.3 pg/ml, respectively.

### Luminex - Th1/Th2 cytokine quantification in supernatant cultures

The Bio-Plex Pro Human Cytokine Th1/Th2 Assay (Bio-Rad Laboratories, Inc) containing fluorescent microspheres conjugated to monoclonal antibodies specific to Th1- and Th2-related cytokines, including IL-2, IL-4, IL-5, IL-10, IL-12, IL-13, GM-CSF, IFN-γ, and TNF, was used.

The cytokine levels in the supernatants of PBMC cultures were analysed according to the manufacturer’s instructions. Briefly, a 50 μl sample obtained from one human and two *Saimiri* (V17 e V31) samples was diluted 1:4 with sample diluent, incubated with antibody-coupled beads and washed. Subsequently, the samples were incubated with biotinylated secondary antibodies, followed by incubation with streptavidin-phycoerythrin. The beads were read on a Luminex System (Bioplex 200, Bio-Rad). The standard curves were generated with a dynamic range between 5 and 20,000 pg/ml, and the data were analysed using Bioplex Manager Software.

A serial dilution of the standards was added to the plate in duplicate to obtain a standard curve. Following the generation of a five-parameter logistic curve, the standard recovery was calculated using the following equation: (observed concentration/expected concentration) × 100. A recovery range between 80 and 120% was used according to the manufacturer’s recommendations. Any sample with a standard recovery value in an area of the curve outside of this range was not considered accurate, and samples were considered positive if the values obtained were above the limits of detection for IL-2, IL-4, Il-5, IL-10, IL-12, IL-13, GM-CSF, IFN-γ and TNF that were 0.003, 0.001, 0.01, 0.006, 0.01, 0.009, 0.004, 0.008 and 0.03 pg/ml, respectively, in accordance with the manufacturer’s recommendations.

### Enzyme-linked immunospot (ELISPOT) cytokine measurements

Several antibodies were used to determine the levels of different cytokines in the culture supernatant, in accordance with methodology previously described [34]. The monoclonal antibodies used in the ELISPOT assays are listed in Table 1.

**Table 1:** **Commercially available human-specific antibodies tested for reactivity with** ***Saimiri*** **cytokines in ELISA and ELISPOT assays**

**Anti-human mAbs**	**Clones**	**Suppliers**	**References**	**Reactivity**	
				**Human**	***Saimiri***
IFN-γ capture	mAb GZ-4	Mabtech	3420 M-3	+	+
IFN-γ biotin	mAb 7-B6-	Mabtech	3420-6	+	+
IFN-γ capture	B27	BD Bioscience	18891D	+	-
IFN- γ biotin	4SB3	BD Bioscience	554550	+	-
IL-4 capture	8D4-8	BD Bioscience	554515	-	-
IL-4 biotin	MP4-25D2	BD Bioscience	18502D	-	-
IL-4 capture	mAb IL4-I	Mabtech	3410-3	+	+
IL-4 biotin	mAb IL4-II	Mabtech	3410-6	+	+
IL-5 capture	TRFK5	BD Bioscience	554393	+	-
IL-5 biotin	JES1-5A10	BD Bioscience	554491	+	-
IL-5 capture	mAb TRFK-5	Mabtech	3490-3	+	-
IL-5 biotin	mAb 5 A10	Mabtech	3490-6	+	-
IL-10 capture	JES3-9D7	BD Bioscience	554497	-	-
IL-10 biotin	JES3-12G8	BD Bioscience	554499	-	-
IL-10 capture	2617KIa	BD Bioscience	51-26171E	+	-
IL-10 biotin	2617KIb	BD Bioscience	51-26172E	+	-
IL-10 capture	mAb 9D7	Mabtech	3430-3	+	-
IL-10 biotin	mAb 12 G8	Mabtech	3430-6	+	-
IL-10 capture	945A5D11	Biosource	AHC8102	+	-
IL-10 biotin	945A5A10	Biosource	AHC7109	+	-
IL-13 capture	mAb IL13 I	Mabtech	3470-3	+	-
IL-13 biotin	mAb IL13	Mabtech	3470-6	+	-
TNFcapture	mAb1	BD Bioscience	554509	+	-
TNF biotin	mAb11	BD Bioscience	18642D	+	-
TNFcapture	2637KIa	BD Bioscience	555212	+	-
TNF biotin	2637KIb	BD Bioscience	555212	+	-

Blood samples from eight *Saimiri sciureus* (U1, R1, O13, O17, Q3, Q7, J3 and J7) and from one clinically healthy human donor were used in ELISPOT assays.

MultiScreen-IP 96-well plates (Millipore) were coated at 4°C overnight with 100 μl of cytokine monoclonal antibody at a concentration of 8 μg/ml in filtered carbonate-bicarbonate buffer (pH 9.6). The plates were subsequently washed three times with sterile PBS, and the uncoated sites were blocked with 200 μl of PBS containing 10% FCS (Imunoquímica) for 2 hrs at 37°C in 5% CO_2_. Approximately 2 × 10^5^ cells/well were transferred to each precoated well and stimulated for 18 hrs with 50 ng/ml PMA (Sigma) and 250 ng/ml ionomycin (Sigma) in complete RPMI 1640 medium containing 15 mM glutamine (Sigma), 10 mM Hepes, 200 U/ml penicillin (Sigma), 200 μg/ml streptomycin (Sigma), 3 mg/ml gentamicin (Sigma) and 2 g/l sodium bicarbonate (Grupo Química) and supplemented with 10% inactivated FCS (Imunoquímica). After three washes with PBS and three washes with PBS/0.05% Tween 20 (PBS/T20 washing buffer), 100 μl of 1 μg/ml diluted biotinylated antibody was added. The plates were incubated for 1 hr at RT and subsequently washed three times with washing buffer. Streptavidin conjugated to horseradish peroxidase (BD Bioscience) was diluted 1:1000 in washing buffer, and 100 μl of this dilution was added to each well. The plates were incubated for 1 hr at RT, followed by washing three times with washing buffer and three times with PBS. The plates were developed using the AEC Substrate Kit (BD Bioscience) comprising 30 ml of AEC chromogen solution with 25 drops of AEC substrate. One-hundred μl of the substrate was added to each well, and the plate was incubated until red spots emerged (10–15 min). Colour development was terminated using washing buffer. The plates were air-dried, and the spot numbers were counted using an Immunospot Series 3B Analyzer (Cellular Technology). The spot count in unstimulated, negative control wells was subtracted from that in the test wells. The mean number of spot-forming units (SFU) in the paired wells was multiplied by four to obtain the SFU per 10^6^ PBMC. Counts ≥20 SFU/10^6^ PBMC and at least ≥ two-fold greater than the background counts were considered positive responses.

### Enzyme-linked immunosorbent assay (ELISA)

ELISA was performed using the same pairs of monoclonal antibodies (Table 1) and blood samples obtained from three *Saimiri sciureus* (B11, J7 and N5) and from one human donor were used in ELISA assay. Briefly, 96-well, flat-bottomed microtitre plates (Nunc) were coated with 100 μl of anti-human monoclonal cytokine detection antibodies at 4 μ g/ml (diluted in 0.1 M NaHCO_3_). After overnight incubation at 4°C, the plates were washed three times with sterile PBS, and the uncoated sites were blocked with 200 μl of PBS containing 10% FCS for 2 hrs at 37°C. After subsequent washing, 100 μl of the supernatant obtained from the culture samples was added to duplicate wells, and the plates were incubated for 2 hrs at 37°C. The plates were subsequently washed, and 100 μl of biotinylated anti-human cytokine detection antibody at 1 μg/ml (1/250 dilution) and 100 μl of streptavidin peroxidase at 2.5 μg/ml. (1/250 dilution) were added. After incubation for 1 hr at 37°C and additional washing, 100 μl/well of orthophenylediamine (OPD) in citrate-phosphate buffer (10 mg/25 ml plus 10 ml H_2_O_2_) (pH 5.0) was added to each well and incubated for 30 min at RT in the dark, followed by the addition of 50 μl/well 2 N H_2_SO_4_ to terminate the reaction. The absorbance was read at 405 nm using a spectrophotometer (Spectra Max 250; Molecular Devices). A standard curve was constructed for each cytokine using different dilutions of human recombinant cytokines.

### Intracellular cytokine secretion (ICS)

Immune cell activation was also determined according to the percentage of cells producing cytokines after *in vitro* stimulation. Blood samples from one *Saimiri* (B11) and from one human donor were used for the evaluation of ICS. Briefly, 1 × 10^6^ purified PBMC/ml were cultured in complete RPMI-1640 medium containing 15 mM glutamine (Sigma), 10 mM Hepes, 200 U/ml penicillin (Sigma), 200 μg/ml streptomycin (Sigma), 3 mg/ml gentamicin (Sigma), and 2 g/l sodium bicarbonate (Grupo Química) and supplemented with 10% inactivated FCS (Imunoquímica) on 24-well plates and incubated for 24 hrs at 37°C, 5% CO_2_. Cells were either cultured in the presence of 100 ng/ml PMA (Sigma) and 500 ng/ml ionomycin (Sigma) or in the absence of mitogen. To block cytokine secretion, the protein transport inhibitor brefeldin A (2.5 μg/ml – BD Bioscience) was added to all samples after 12 hrs of culture. After 24 hrs, the cells were harvested and washed with PBS. Another incubation with 100 μl of permeabilization solution (PBS/0.2% saponin/10% FCS/1% bovine serum albumin (BSA) was performed at RT. After centrifugation at 1,500 rpm for 10 min, the cells were subsequently incubated for 30 min at RT with anti-human monoclonal cytokine detection or capture antibodies (Table 2) previously diluted in permeabilization solution. After two washes with PBS/10% FCS/1% BSA, the cells were incubated for 30 min with anti-mouse IgG FITC-labeled in permeabilization solution. The cells were washed twice with PBS/0.2% saponin and once with PBS and then resuspended in 2% paraformaldehyde. The experiments were analysed using a FACSCalibur flow cytometer (FACSCalibur, BD Biosciences). Over 100,000 lymphocyte-gated events were collected.

**Table 2:** **Commercially available human-specific antibodies tested for reactivity with** ***Saimiri*** **cytokines in ICS assays**

**Reagents**	**Clones**	**Suppliers**	**References**	**Human**	***Saimiri***
IFN-γ capture	mAb GZ-4	Mabtech	mAb GZ-4	+	+
IFN-γ biotin	mAb 7-B6-1	Mabtech	mAb 7-B6-1	+	+
IFN-γ capture	B27	BD Bioscience	18891D	+	-
IFN-γ biotin	4SB3	BD Bioscience	554550	+	-
IL-4 capture	8D4-8	BD Bioscience	554515	-	-
IL-4 biotin	MP4-25D2	BD Bioscience	18502D	-	-
IL-5 capture	TRFK5	BD Bioscience	554393	+	-
IL-5 biotin	JES1-5A10	BD Bioscience	554491	+	-
IL-10 capture	JES3-9D7	BD Bioscience	554497	-	-
IL-10 biotin	JES3-12G8	BD Bioscience	554499	-	-
IL-10 capture	945A5D11	Biosource	AHC8102	-	-
IL-10 biotin	945A5A10	Biosource	AHC7109	-	-
IL-13 capture	mAb IL13-I	Mabtech	3470-3	+	-
IL-13 biotin	mAb IL13-II	Mabtech	3470-6	+	-
TNF capture	mAb1	BD Bioscience	554509	+	-
TNF biotin	mAb11	BD Bioscience	18642D	+	-

### Analysis of gene expression using TaqMan Array Human Cytokine Assays for real-time PCR

The gene expression of IL-1, IL-2, IL-3, IL-4, IL-5, IL-6, IL-8, IL9, IL-10, IL-12, IL-13, IL-15, IL-16, IL-17, IL-18, IFN-α, IFN-β, IFN-γ, LTA, TNF, the 18S control, and three candidate endogenous control genes (GAPDH, HPRT1 and GUSB) was assessed through real-time PCR using TaqMan® Gene Expression Array Plates (Applied Biosystems) containing predesigned, gene-specific primers and probes (Table 3). Additional primers and probes were designed based on the published *Saimiri sciureus* sequences for IL-4, IL-5, IL-6, LTA and IFN-γ (IL-4: GenBank DQ985388.1; IL-5: GenBank AF294756.1; IL-6: GenBank AF294757.1 and LTA: GenBank FJ589021.1). The gene expression of IL-4, IL-5, IL-6 and LTA was also assessed through real-time PCR using Custom TaqMan® Gene Expression Assays (Table 3).

**Table 3:** **Assays identifications for the genes used in real-time PCR using inventoried and custom TaqMan Gene Expression Assays**

**Gene**	**ID**
Inventoried Assays	
18S	Hs99999901_s1
GAPDH	Hs99999905_m1
HPRT1	Hs99999909_m1
GUSB	Hs99999908_m1
IFNA1	Hs00855471_g1
IFNA16	Hs03005057_sH
IFNA17	Hs00819693_sH
IFNA2	Hs02621172_s1
IFNA6	Hs00819627_s1
IFNA7	Hs01652729_s1
IFNA8	Hs00932530_s1
IFNB1	Hs00277188_s1
IFNG	Hs00989291_m1
IL-10	Hs00961622_m1
IL-12A	Hs01073447_m1
IL-12B	Hs01011518_m1
IL-13	Hs99999038_m1
IL-15	Hs01003716_m1
IL-16	Hs00189606_m1
IL-17A	Hs00174383_m1
IL-18	Hs99999040_m1
IL-1A	Hs00174092_m1
IL-1B	Hs01555410_m1
IL-2	Hs00174114_m1
IL-3	Hs00174117_m1
IL-4	Hs00174122_m1
IL-5	Hs00174200_m1
IL-6	Hs00985639_m1
IL-8	Hs99999034_m1
IL-9	Hs00174125_m1
LTA	Hs00236874_m1
TNF	Hs00174128_m1
Custom Assays	
IL-4	AIWR2WR
IL-5	AIVI4QJ
IL-6	AIT96KB
LTA	AIS08D3
IFNG	AIRR97V

In order to evaluate the analysis of gene expression of cytokines, it was used a total of nine *Saimiri* (V17, V31, 24, 149, 51, S8, S34, S36 and PA51) and one human blood samples. RNA was extracted from triplicate wells of PBMC and splenocytes using the PureLink RNA Mini Kit (Ambion, Inc.) according to the manufacturer’s instructions. Reverse transcription was performed using High Capacity cDNA Reverse Transcription (Applied Biosystems). Briefly, 10 μl of each extracted RNA was added to 10 μl of reaction mix containing 2 μl of 10x RT buffer, 0.8 μl of 25X dNTP Mix, 2 μl of 10X RT Random Primers, 1 μl of MultiScribe™ Reverse Transcriptase and 4.2 μl of nuclease-free water. The thermal cycling conditions consisted of 25°C for 10 min, 37°C for 120 min and 85°C for 5 min.

Each well of the TaqMan® Array Plate was reconstituted using 10 μl of TaqMan® Gene Expression Master Mix (Applied Biosystems) and 10 μl of template cDNA. For custom assays, 9 μl of template cDNA was added in a 20-μl reaction volume containing 1 μl of the 20x TaqMan Gene Expression Assay Mix and 10 μl of the 2x TaqMan Gene Expression Master Mix. The reaction was performed using an Applied Biosystems real-time PCR 7500 System with the following cycling conditions: 50°C for 2 min and 95°C for 10 min, followed by 40 cycles of 95°C for 15 sec and 60°C for 60 sec. The calculation method used for relative quantification was the comparative CT method. The relative expression was determined as the expression of the corresponding genes in PBMC obtained from the healthy human donor or of the corresponding genes in unstimulated PBMC or splenocytes cultures.

## Results

In the present study, the gene expression of cytokines from *Saimiri* was analysed in PBMC stimulated with PMA and ionomycin. The cross-reactivity of human cytokine antibodies for cytokines secreted by *Saimiri* cells was evaluated using Luminex, CBA, ELISA, ELISPOT, and ICS. For all employed assays, the mononuclear cells were stimulated using mitogens, and human cells or the supernatants obtained from human cells were used as controls.

### Human and non-human BD cytometric bead array Th1/Th2 cytokine kit

**Figure 1:**
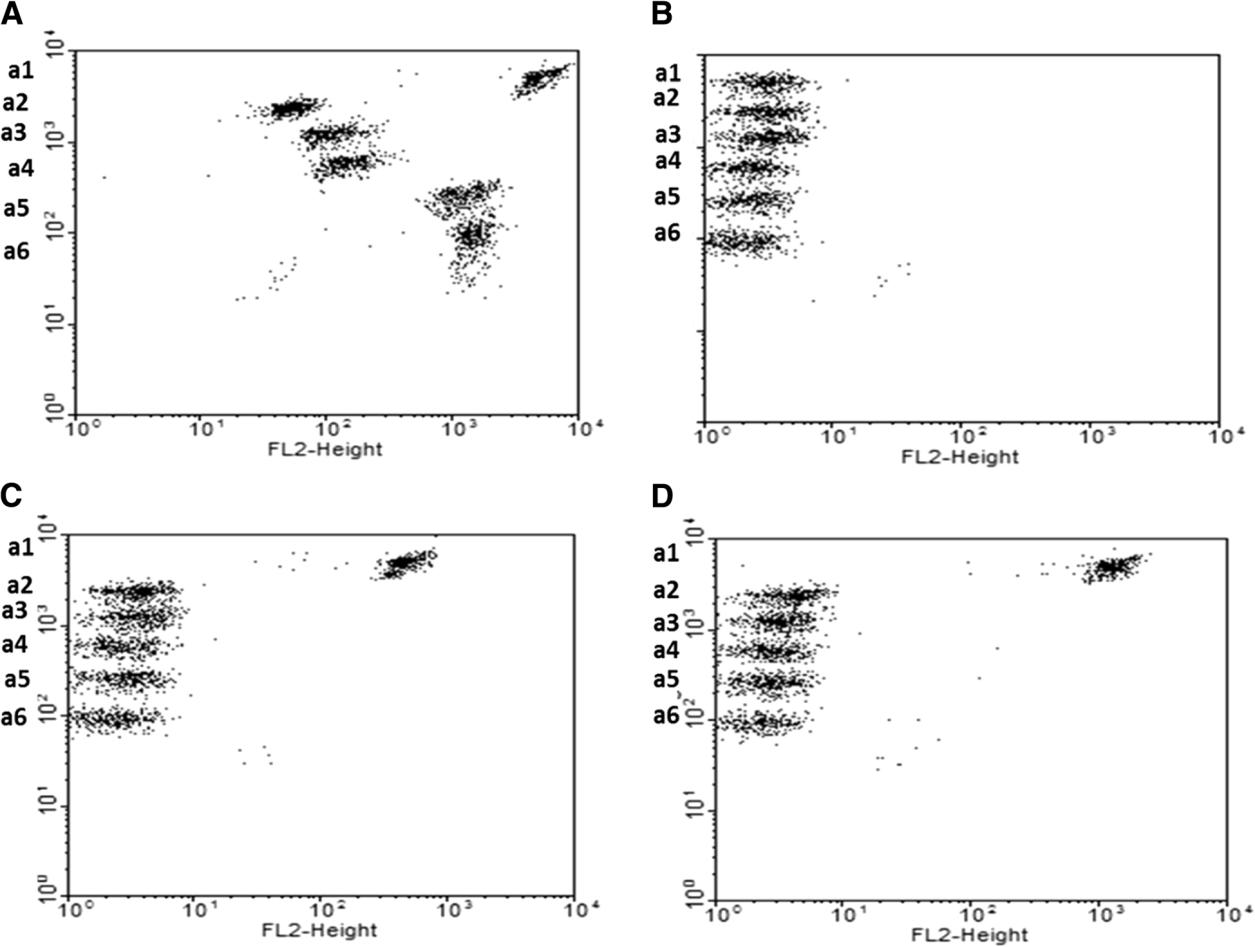
Dotplots of cytokine in the supernatants of PBMC using CBA kit. Supernatant from PBMC cultured in presence or absence of stimulus obtained from **(A)** a healthy donor in the presence of PMA/ionomycin; **(B)** *Saimiri* V17 in the absence of stimulus; **(C)** *Saimiri* V17; and, **(D)** *Saimiri* V31 in the presence of mitogen. a1 (IL-2), a2 (IL-4), a3 (IL-6), a4 (IL-10), a5 (TNF), and a6 (IFN-γ).

The CBA assays were used to analyse the secretion of cytokines (IL-2, IL-4, IL-5, IL-6, IL-10, TNF, and IFN-γ) in PBMC cultures from five *Saimiri* monkeys and one human donor. As expected, upon *in vitro* stimulation of human mononuclear cells with PMA/ionomycin, increased cytokine secretion was observed in the supernatants of stimulated cells compared to unstimulated cultures (Figure 1). However, the use of both human and non-human CBA kits facilitated the detection of only IL-2 in the supernatants of cell cultures from *Saimiri*, even after 6 hrs of stimulation with mitogens (Figure 1).

### Luminex

**Figure 2:**
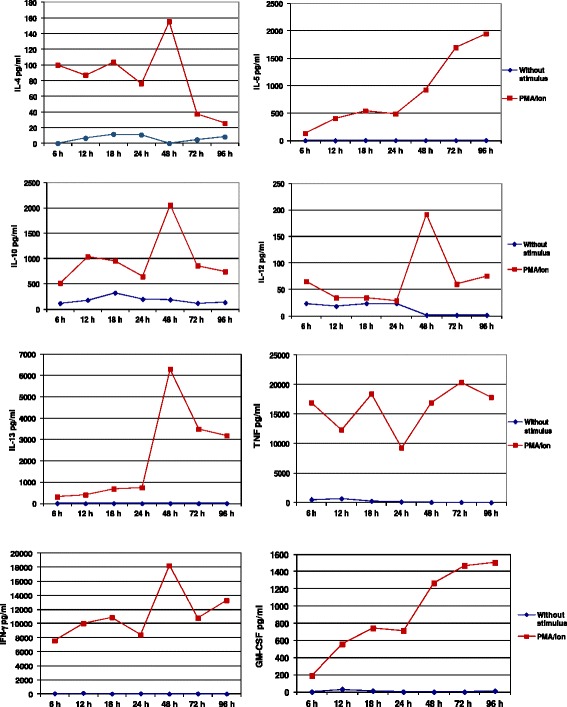
Cytokine concentrations in the supernatant of PBMC using the Luminex assay. The samples were obtained from PBMC isolated from a healthy human donor and cultured in the presence or absence of mitogenic stimulus.

**Figure 3:**
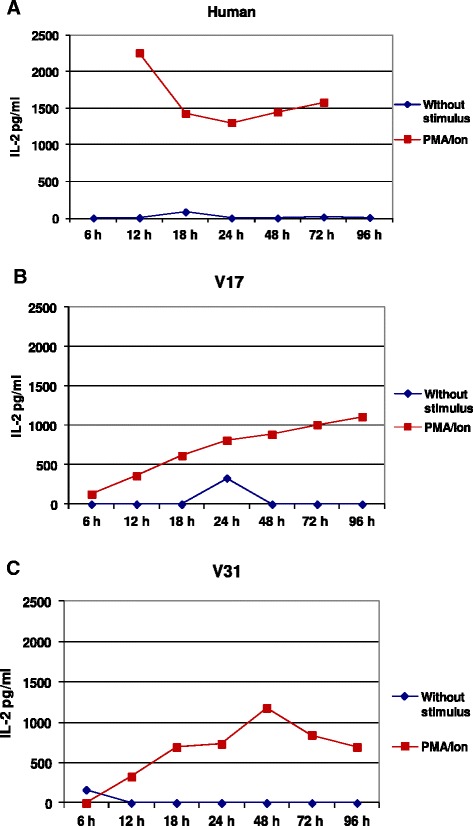
Detection of IL-2 in the supernatant of PBMC from *Saimiri sciureus* using Luminex assay. IL-2 concentration in the supernatant of cells cultured in the presence or absence of mitogenic stimulus from **(A)** a healthy human donor and **(B and C)** two *Saimiri sciureus* (V17 and V31) samples.

The ability of Bio-Plex Pro Human Cytokine Th1/Th2 Assay Kit to detect S. *sciureus* IL-2, IL-4, IL-5, IL-10, IL-12, IL-13, GM-CSF, IFN-γ and TNF cytokines was tested using supernatant samples from PBMC isolated from two monkeys and one healthy donor after stimulation with PMA/ionomycin for 6, 12, 18, 24, 48, 72 and 96 hrs. As expected, Luminex assay was able to detect all evaluated cytokines in the supernatants from mitogenic stimulated-human PBMC (Figures 2 and 3). The higher responses for each cytokine were: 12 hrs for IL-2; 48 hrs for IL-4, IL-10, IL-12, IL-13 and IFN-γ; 72 hrs for TNF and; 96 hrs for IL-5 and GM-CSF. In contrast, only IL-2 was detected in the supernatant of PBMC cultures isolated from *Saimiri* V31 and V17, showing higher levels after 48 hrs and 96 hrs, respectively (Figure 3).

### ELISA and ELISPOT assays

The cross-reactivity of anti-human cytokines antibodies with cytokine produced by *Saimiri* stimulated PBMC was also evaluated by ELISA and ELISPOT.

**Figure 4:**
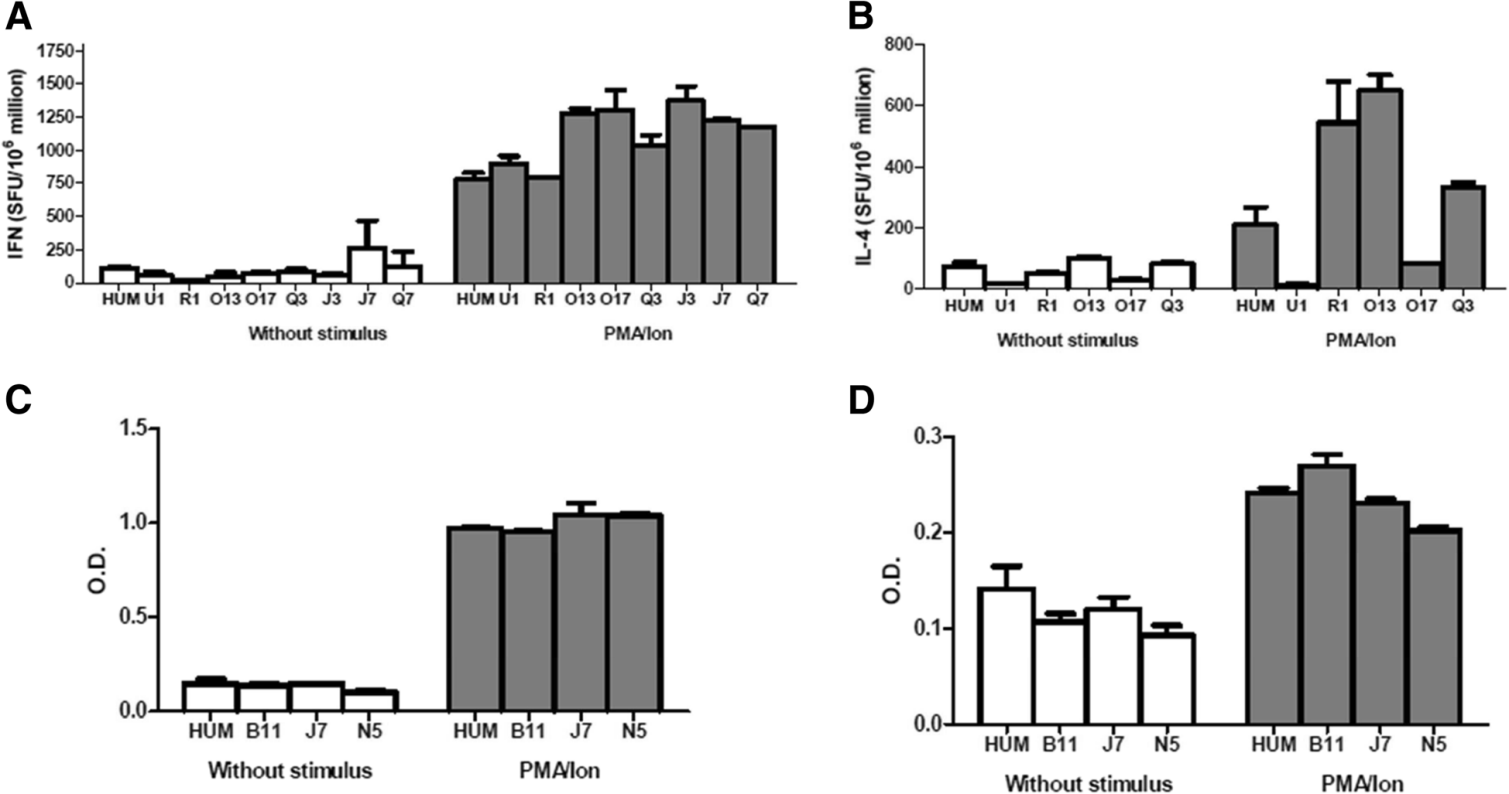
Results of the IFN-γ and IL-4 responses to PMA/ionomycin activation of PBMC from *Saimiri sciureus* samples. Samples from nine *Saimiri* (U1, R1, O13, O17 Q3, J3, J7 B11 and N5) and one clinically healthy human donor (HUM) were cultured in absence or presence of PMA/ionomycin (PMA/Ion) for 18 hrs. The results of ELISPOT for **(A)** IFN-γ and **(B)** IL-4 are presented as the mean of the number of SFU/10^6^ cells ± SD. Mean of OD values obtained ELISA assays for **(C)** IFN-γ and **(D)** IL-4 ± SD.

ELISA and ELISPOT assays for the detection of TNF, IFN-γ, IL-4, IL-5, IL-10, and IL-13 were performed using fresh PBMC isolated from ten *Saimiri* monkeys and one healthy human donor. Table 1 shows the information for the human-specific antibodies used to test for reactivity in *S. sciureus* cells. In both ELISPOT and ELISA assays, only anti-human antibodies from Mabtech suppliers facilitated the measurement of IFN-γ (mAb GZ-4 and 7-B6-1 clones) and IL-4 (mAb IL4-I and mAb IL4-II clones) in both PBMC cultures and the supernatant obtained from cultured S. *sciureus* cells (Table 1). The results containing the numbers of SFU in ELISPOT assays and the optical densities (O.D.) values obtained in ELISA for IFN-γ and IL-4 are represented in Figure 4.

### ICS assay

The ICS assay was performed using human monoclonal antibodies for IL-4, IL-5, IL-10, IL-13, IFN-γ, and TNF and the positivity was determinate setting the region of cytokine-producing lymphocytes using mitogen stimulated and non-stimulated human PBMC cultures. However, only the anti-IFN-γ (clones mAb GZ-4 and 7-B6-1) human antibodies from Mabtech supplier facilitated the detection of this cytokine in both human and NHP cells (Table 2).

### Gene expression using real-time PCR

**Figure 5:**
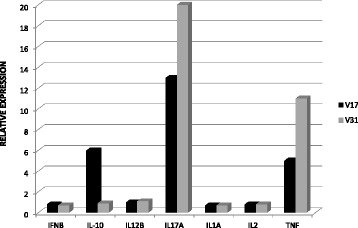
Relative expression of cytokines in PBMC from *Saimiri sciureus* using TaqMan® Gene Expression Array Plates. Gene expression analysis of IFN-γ, IL-10, IL-12B, IL-17A, IL-1A, IL-2 and TNF in PBMC from two *Saimiri sciureus* (V17 and V31) cultured in the presence of PMA/ionomycin for 12 hours. The relative expression was determined as the expression of the corresponding genes in PBMC obtained from the healthy human donor.

In the first experiment, PBMC from two *Saimiri* (V17 and V31) samples and one human sample were cultured in the presence or absence of PMA/ionomycin for 6, 12, 24, 48 and 72 hrs. Gene expression analysis was performed using TaqMan® Gene Expression Array Plates. A total of 12 of the 28 cytokines assayed were detected in NHP samples, including IL-1A, IL-2, IL-4, IL-5, IL-6, IL-10, IL-12B, IL-17, IFN-β, IFN-γ, LTA, TNF, and two housekeeping genes. However, only seven cytokines (IL-1A, IL-2, IL-10, IL-12B, IL-17, IFN-β, and TNF) presented relative expression of at least 70% of the corresponding genes in PBMC culture obtained from the healthy human donor (Figure 5). As expected, the gene expression of the 28 cytokines and four housekeeping genes in PBMC from the human donor was readily detected.

**Figure 6:**
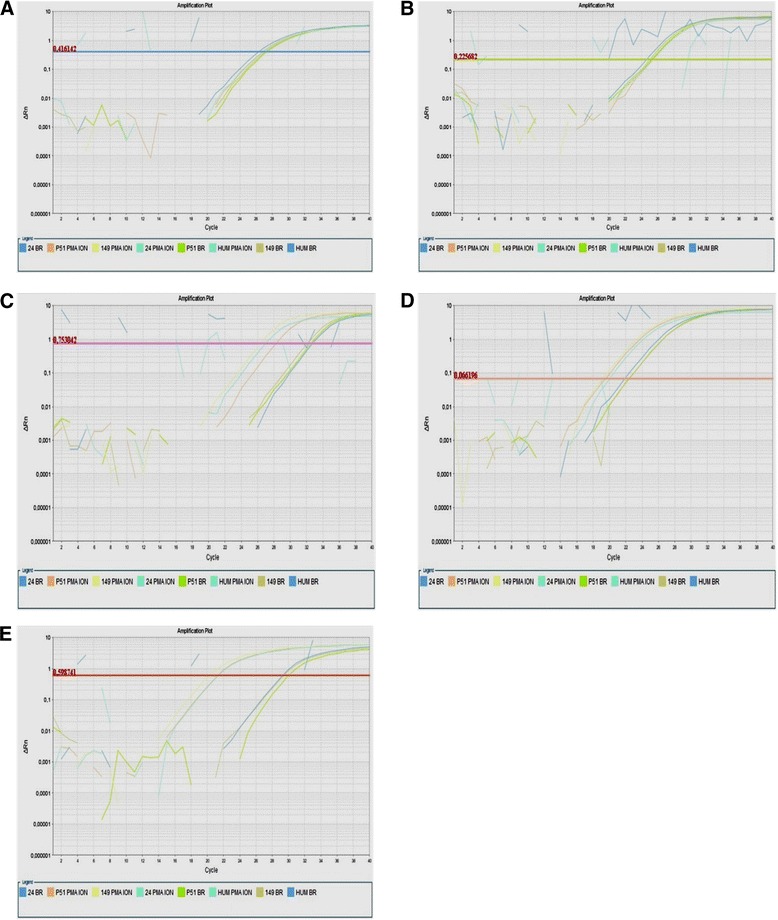
Plots showing real-time PCR amplification using custom TaqMan® Gene Expression Assays. Samples from three *Saimiri sciureus* (24, P51 and 149) and one human health donor (HUM) were cultured in absence (BR) or presence of PMA/ionomycin (PMA ION) for 12 h. Delta Rn versus cycle amplification plots were obtained using primers and probes for **(A)** IL-4, **(B)** IL-5, **(C)** IL-6, **(D)** LTA, and **(E)** IFN-γ. The lines represent the threshold.

In the second experiment, PBMC from three *Saimiri* (24, 149 and 51) samples and PBMC obtained from one human blood sample were cultured in the presence or absence of PMA/ionomycin for 12, 24 and 48 hrs. Gene expression analysis using primers and probes for IL-4, IL-5, IL-6, LTA, and IFN-γ was performed using the Custom TaqMan Gene Expression Assay with primer sets specifically designed based on *Saimiri* sequences. The results revealed the gene expression levels of the five cytokines in PBMC from *Saimiri*, whereas no gene expression was detected in human PBMC (Figure 6).

**Figure 7:**
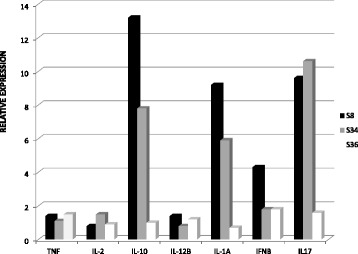
Relative expression of cytokines in PBMC from *Saimiri sciureus* using TaqMan® Gene Expression Array Plates. The gene expression analysis of IFN-γ, IL-10, IL-12B, IL-17A, IL-1A, IL-2 and TNF from three *Saimiri sciureus* (S8, S34 and S36) cultured in the presence of PMA/ionomycin for 12 hours. The relative expression was considered as the expression of the corresponding genes in PBMC obtained from the healthy human donor.

**Figure 8:**
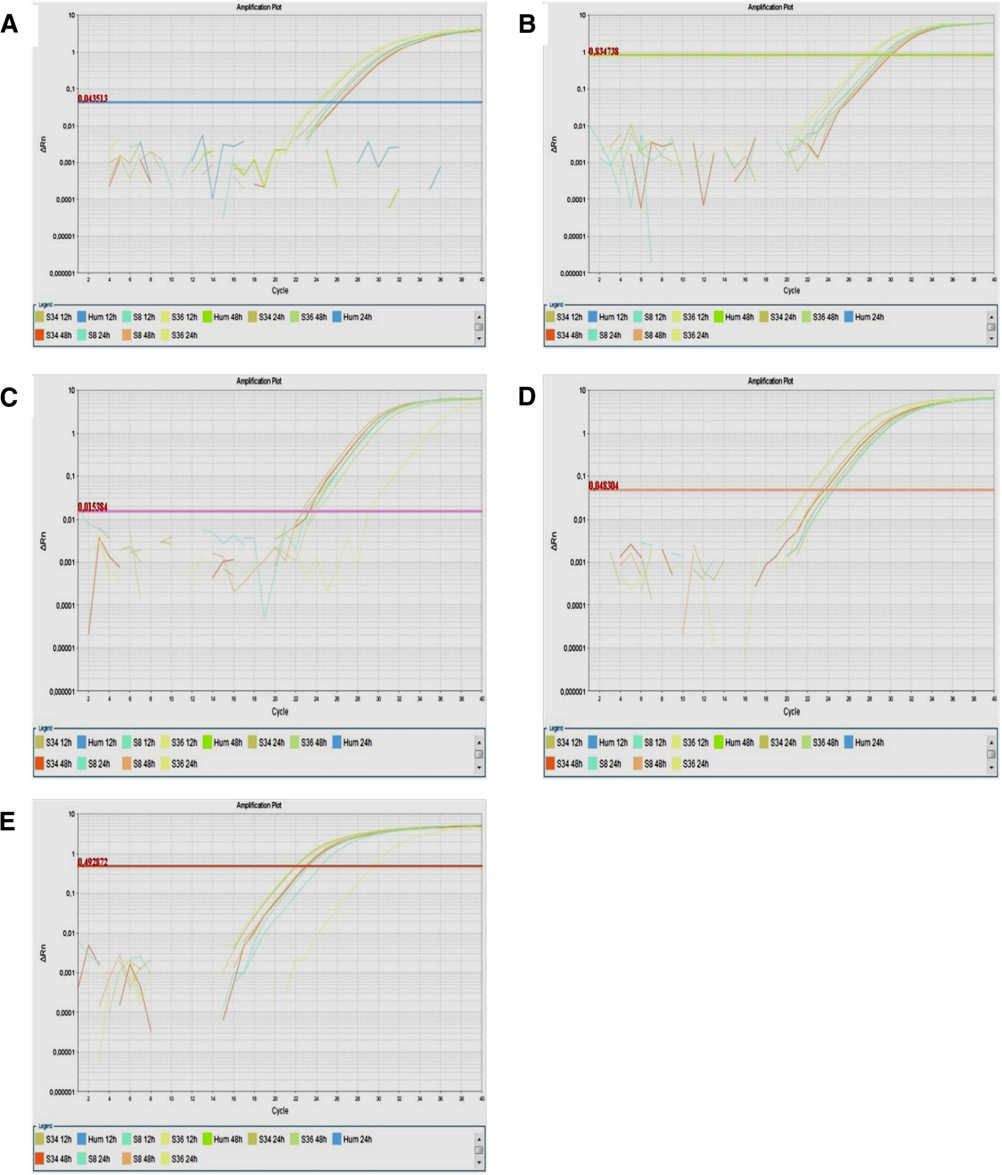
Plots showing real-time PCR amplification using custom TaqMan® Gene Expression Assays. Samples from three *Saimiri sciureus* (S8, S36 and S34) and one healthy human donor (HUM) were cultured in absence (BR) or presence of PMA/ionomycin (PMA ION) for 12 h, 24 and 48 h. Delta Rn versus cycle amplification plots were obtained using primers and probes for **(A)** IL-4, **(B)** IL-5, **(C)** IL-6, **(D)** LTA, and **(E)** IFN-γ. The lines represent the threshold.

In order to confirm the results obtained in the first and second experiments, in the third experiment, PBMC from three *Saimiri* (S8, S34 and S36) samples and from one human donor sample were cultured in the presence or absence of PMA/ionomycin for 12, 24 and 48 hrs. Gene expression of IFN-γ, IL-10, IL-12B, IL-17A, IL-1A, IL-2 and TNF was assessed using TaqMan Gene Expression Plates and the gene expression of IL-4, IL-5, IL-6, LTA, and IFN-γ was assessed using Custom TaqMan Gene Expression Assay. The TaqMan Gene Expression Plates facilitated the detection of the gene expression of IL-1A, IL-2, IL-10, IL-12B, IL-17, IFN-β, and TNF in PBMC from *Saimiri*, showing a relative expression of at least 70% of the gene expression observed in the PBMC obtained from the human donor in the presence of PMA/ionomycin (Figure 7). Similarly, the Custom TaqMan Gene Expression Assay revealed the gene expression of IL-4, IL-5, IL-6 LTA, and IFN-γ, although expression of these genes was not detected in human PBMC (Figure 8).

**Figure 9:**
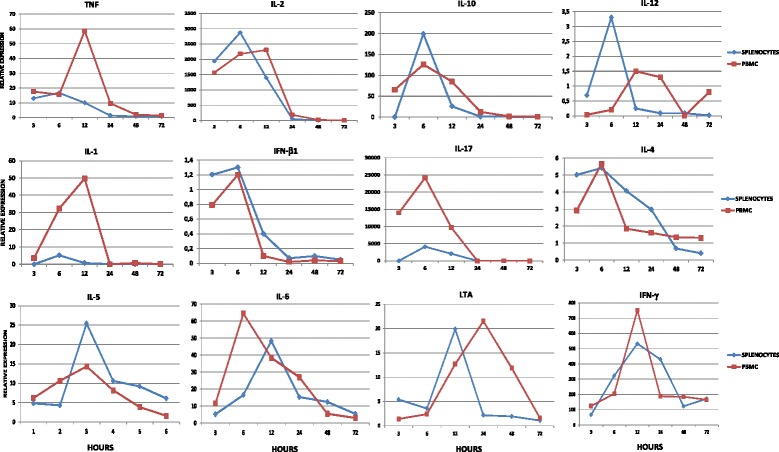
Kinetics of cytokine gene expression in PBMC and splenocytes. The cells were obtained from one *Saimiri sciureus* (PA51) and cultured for 3, 6, 12, 24, 48, and 72 hrs in the absence or presence of PMA/ionomycin. The relative expression was considered as the expression of the corresponding genes in PBMC or splenocyte cultured in absence of PMA and ionomycin.

In the fourth experiment, PBMC and splenocytes obtained from one *Saimiri* (PA51) sample were cultured in the presence or absence of PMA/ionomycin for 3, 6, 12, 24, 48 and 72 hrs, and the gene expression was analysed using TaqMan Gene Expression Plates and the Custom TaqMan Gene Expression Assay. The relative expression was determined as the expression of the corresponding genes in unstimulated PBMC or splenocytes cultures. Gene expression of IL-1A, IL-2, IL-10, IL-12B, IL-17, IFN-β, and TNF was detected using the plates, and the Custom TaqMan Gene Expression Assay revealed the gene expression of IL-4, IL-5, IL-6 LTA, and IFN-γ in both PBMC and splenocytes from *Saimiri*. Furthermore, the results showed that the optimal time point for the evaluation of gene expression for the studied cytokines was at 6–12 hrs after culture, with the exception of LTA in PBMC, which exhibited optimal expression after 24 hrs of culture (Figure 9).

## Discussion

Malaria remains a serious public health problem and the most important parasitic disease in developing countries in tropical regions [35]. The development of an effective vaccine represents an important measure for disease control, as this treatment approach would be preventative. For effective vaccine development, it is essential to understand the mechanisms underlying the acquisition of malaria immunity in humans [36-39]. However, studies in humans are complex and beyond ethical limitations [40,41], and many other limitations, such as the lack of access to relevant organ and tissue samples and the difficulty to manipulate the immune response, make the use of experimental models necessary. Therefore, the use of experimental models phylogenetically related to humans is ideal. Indeed, the WHO has recommended the use of neotropical primates of the genus *Aotus* and *Saimiri* for experimental studies on malaria [17].

Notwithstanding these benefits, there are restrictions to the use of these animals, and the lack of specific reagents represents an important constraining factor. Therefore, the development and use of immunological and molecular tools could facilitate the reliable evaluation of the immune response in preclinical trials of malaria vaccines and mediate the characterization of the pathogenesis of disease in these animals [42-45].

Cytokines secreted from cells of the immune system play essential roles during malaria [46]. Thus, specific reagents and assays for the detection and quantification of these molecules could provide information concerning the immunological and physio-pathological changes that occur during experimental infection of these animals.

Other studies involving *Saimiri* and *Aotus* have previously been conducted for the development of molecular reagents. Several genes encoding molecules involved in the immune response, such as cytokines, Duffy blood factor, T cell immunoglobulin receptor, and Toll-like receptors, have been sequenced, and these results have shown greater than 80% identity to the human counterparts. For instance, degrees of identity between 91.4 and 98.1% for the sequences of IL-1β, IL-2, IL-5, IL-6, IL-10, IFN-γ, and TNF from S. *sciureus* and other NHP and humans were observed [30]. These data suggest that human sequences as templates for the design of *Saimiri*-specific primers and probes may be used to obtain amplification products to study cellular immune responses in *Saimiri* and *Aotus* [33,47,48].

In the present study, molecular and immunological tools were developed to evaluate the immune response of the NHP S. *sciureus* using human and non-human CBA, Luminex, ELISA, ELISPOT, ICS, and real-time PCR assays.

The use of five assays specific for humans (CBA, Luminex, ELISA, ELISPOT and ICS) facilitated the detection of a limited number of cytokines in NHP, including IL-2 in humans and NHP with CBA and Luminex, IL-4 and IFN-γ with ELISPOT and ELISA and IFN-γ with ICS. These findings may reflect the reduced specificity of human monoclonal antibodies to *Saimiri* cytokines. A previous study reported difficulty in detecting TNF from Old World monkeys using commercial enzyme-linked immunoassay kits designed for the detection and quantification of human TNF [49]. It was suggested that the 62- to 72-amino acid region of TNF might contribute to the specificity of monoclonal antibodies to this cytokine in humans. Furthermore, this region in the *Saimiri* sequence is highly divergent, with six substitutions and the addition of a glutamine residue at position 62. These alterations may reduce the cross-reactivity of monoclonal antibodies with *Saimiri* TNF, a fact that should be considered when using this antibody for the detection and quantification of TNF from *Saimiri sciureus*. The effect of small sequence differences in the recognition of orthologous molecules in different species has also been illustrated in studies showing that the injection of recombinant human cytokines in NHP induced potent neutralizing antibody responses against these cytokines, even considering the high homologies (>95%) in the peptide and nucleotide sequences [28]. Differences in the sequences and conformations might also explain the results previously obtained by using a panel of 67 monoclonal antibodies from different manufacturers to define the reactivity of these molecules against CD markers in *Saimiri* mononuclear cells [50]. In some cases, adequate reactivity was observed, with variation in the intensity of staining depending on the clone and manufacturer used, although in many cases no reactivity was detected. Similarly, one study tested 204 antibodies against various human CD markers, and only 28% of these molecules reacted with *Aotus* splenocytes [51]; approximately 40% of the reacting antibodies also recognized rhesus and cynomolgus cells. The use of NHP CBA only facilitated the detection of IL-2, reflecting the broader specificity of antibodies to human cytokines that also cross-react with rhesus, cynomolgus, baboon and pig-tailed macaque cytokines. These findings highlight the need for the development of immunological tools for cytokine detection in *S. sciureus*.

Gene expression of cytokines in cell cultures from *S. sciureus* was evaluated. First, gene expression analysis was performed using TaqMan® Gene Expression Array Plates containing predesigned primers and probes for the detection of 28 human cytokine transcripts. These results revealed the detection of 12 (IL-1A, IL-2, IL-4, IL-5, IL-6, IL-10, IL-12B, IL-17, IFN-β, IFN-γ, LTA, and TNF) of the 28 cytokines and two housekeeping genes. However, only seven (IL-1A, IL-2, IL-10, IL-12, IL-17, IFN-β, and TNF) of these cytokines presented a relative quantification of at least 70% of the gene expression observed in human cells after mitogenic stimulation. The failure in the amplification of some cytokines may reflect the partial similarity between sequences in *Saimiri* and the corresponding genes in humans [33]. However, when comparing the mRNA sequences for the cytokines from *S. sciureus* obtained from GenBank with human sequences using the Basic Local Alignment Search Tool (BLAST), similar degrees of identity between the amplified and non-amplified cytokines were observed. For example, the degree of identity for the genes encoding IL-4 (not amplified) and TNF (amplified) between humans and *S. sciureus* was 92%. Thus, the failure in the amplification of some cytokines most likely reflects small changes in the existing sequences between the human gene and the corresponding *Saimiri* gene, leading to an imperfect annealing of the primer to the DNA template.

Among the 12 cytokines initially amplified, five (IL-4, IL-5, IL-6, LTA, and IFN-γ) showed relative expression levels below the threshold of 70% of the gene expression observed in human cells. However, when primers and probes specific for *S. sciureus* were designed from the published sequences in GenBank, it was possible to detect these cytokine transcripts in mononuclear cells from *S. sciureus*, highlighting the need for the design and use of specific reagents for use with these models, as small differences in sequences can result in difficulties or even preclude proper annealing.

Moreover, PBMC and splenocytes from one *S. sciureus* sample were cultured in the presence or absence of stimuli classically used to stimulate cells *in vitro* (PMA/ionomycin) since Ag specific stimuli would not be broad enough to a detectable activation of cells for 3, 6, 12, 24, 48, and 72 hrs to evaluate the kinetics of IL-1A, IL-2, IL-4, IL-5, IL-6, IL-10, IL-12B, IL-17A, TNF, IFN-β, LTA, and IFN-γ expression. The results demonstrated the gene expression of these cytokines in both PBMC and splenocytes cultures. Furthermore, the results showed that the cytokine transcripts were detected after 6 and 12 hrs of culture, showing the detection of IL-2, IL-6, IL-10, IL-12, IFN-γ, TNF, and LTA transcripts after 6 hrs of *Saimiri* PBMC culture in the presence of PMA/ionomycin. These results are in agreement with a previous report showing similar patterns of gene expression of these cytokines upon PMA/ionomycin stimulation of *Saimiri* splenocytes using *Saimiri*-specific primers in SYBR-green-based real time PCR assays [52].

Unfortunately, cytokines IL-1B, IL-3, IL-8, IL-9, IL-12A, IL-13, IL-15, IL-16, IL-18, IFN-α1, IFN-α2, IFN-α6, IFN-α7, IFN-α8, IFN-α16, or IFN-α17 were not successfully amplified. Amplification of these genes therefore requires additional effort to define primers and probes, as the sequences for these genes in *S. sciureus* have not been published.

## Conclusions

In the present study, a variety of techniques to extend the range of reagents available for NHP research, with the aim of facilitating a more thorough evaluation of the immune response in the *Saimiri sciureus* experimental model of human *Plasmodium* infection was assessed. The data presented herein validate the approach used for the extension of the available reagent range, facilitating a more thorough evaluation of the immune response in this model.
